# Male Germ Cell-Specific RNA Binding Protein RBMY: A New Oncogene Explaining Male Predominance in Liver Cancer

**DOI:** 10.1371/journal.pone.0026948

**Published:** 2011-11-04

**Authors:** Daw-Jen Tsuei, Po-Huang Lee, Hsiao-Yu Peng, Shau-Lin Lu, De-Shiuan Su, Yung-Ming Jeng, Hey-Chi Hsu, Shu-Hao Hsu, Jia-Feng Wu, Yen-Hsuan Ni, Mei-Hwei Chang

**Affiliations:** 1 Department of Pediatrics, College of Medicine, National Taiwan University, National Taiwan University Hospital, Taipei, Taiwan; 2 Department of Surgery, College of Medicine, National Taiwan University, National Taiwan University Hospital, Taipei, Taiwan; 3 Department of Pathology, College of Medicine, National Taiwan University, National Taiwan University Hospital, Taipei, Taiwan; 4 Hepatitis Research Center, College of Medicine, National Taiwan University, National Taiwan University Hospital, Taipei, Taiwan; University of Texas Southwestern Medical Center at Dallas, United States of America

## Abstract

Male gender is a risk factor for the development of hepatocellular carcinoma (HCC) but the mechanisms are not fully understood. The RNA binding motif gene on the Y chromosome (RBMY), encoding a male germ cell-specific RNA splicing regulator during spermatogenesis, is aberrantly activated in human male liver cancers. This study investigated the *in vitro* oncogenic effect and the possible mechanism of RBMY in human hepatoma cell line HepG2 and its *in vivo* effect with regards to the livers of human and transgenic mice. RBMY expression in HepG2 cells was knocked down by RNA interference and the cancer cell phenotype was characterized by soft-agar colony formation and sensitivity to hydrogen-peroxide-induced apoptosis. The results revealed that RBMY knockdown reduced the transformation and anti-apoptotic efficiency of HepG2 cells. The expression of RBMY, androgen receptor (AR) and its inhibitory variant AR45, AR-targeted genes insulin-like growth factor 1 (IGF-1) and insulin-like growth factor binding protein 3 (IGFBP-3) was analyzed by quantitative RT-PCR. Up-regulation of AR45 variant and reduction of IGF-1 and IGFBP-3 expression was only detected in RBMY knockdown cells. Moreover, RBMY positive human male HCC expressed lower level of AR45 as compared to RBMY negative HCC tissues. The oncogenic properties of RBMY were further assessed in a transgenic mouse model. Liver-specific RBMY transgenic mice developed hepatic pre-cancerous lesions, adenoma, and HCC. RBMY also accelerated chemical carcinogen-induced hepatocarcinogenesis in transgenic mice. Collectively, these findings suggest that Y chromosome-specific RBMY is likely involved in the regulation of androgen receptor activity and contributes to male predominance of HCC.

## Introduction

Hepatocellular carcinoma (HCC) is one of the leading cancers in the world [1]. The major identified risk factors include hepatitis B virus (HBV) or hepatitis C virus (HCV) infection, exposure to aflatoxins, and male gender [2]. The male-to-female ratio of HCC reportedly averages 4–5∶1 and is particularly higher in HBV-related HCC (5–11∶1) [3]. Epidemiological studies showed that an elevated level of serum testosterone was significantly associated with an increased risk of HCC in male HBV carriers [4]. However, male predominance with a ratio of 3–4∶1 is also observed in childhood HCC with early onset, as young as <10 years of age before puberty, and cannot be explained directly by androgen effect [5], [6].

The androgen receptor (AR) has been shown to contribute to the male preference of hepatocarcinogenesis in HBV and HCV carriers [7], [8]. The AR protein mediates the action of androgens and can promote the development of male HCC in mice [9]. After binding to ligands, AR forms homodimers and activates the transcription of target genes such as insulin-like growth factor 1 (IGF-1) and insulin-like growth factor binding protein 3 (IGFBP-3) [10], [11]. The AR function is regulated by co-activators or co-repressors [12], the inhibitory isoform AR45 [13], and shorter CAG repeats (that leads to higher AR activity) in the first exon of the AR gene [14]. The HBV X protein is a well-known AR co-activator [15], [16] and was reported to contribute to male predominance in HBV-related human male HCC [17]. In non-HBV HCC, other unknown gender-specific factors promoting HCC formation in males have become a major concern.

Dysregulation of Y chromosome-specific genes has been found in male HCC, but their roles in the male predominance of HCC so far have not been addressed [18], [19]. The RNA-binding motif gene on the Y chromosome (RBMY) is a male germ cell-specific expression gene containing RNA recognition motifs at the N terminus [20]. Its expression is confined to the nuclei of male germ cells and its deletion may cause the arrest of germ cells at the meiotic stage of spermatogenesis [21]. RBMY functions as a male germ cell-specific splicing regulator by modulating the activity of constitutively expressed splicing factors [22], [23]. Its aberrant activation is detected in about 1/3 of male HCC and hepatoblastoma tumor tissues, but not in paired non-tumor liver tissues, female HCC, or other types of cancers [24]. Though RBMY can interact with AR co-activator Sam68 [25], [26], its role in the AR signaling pathway has not yet been reported.

The present study aimed to evaluate the transformation and anti-apoptotic efficacy of RBMY in human hepatoma cells, and the hepatocarcinogenic efficacy of RBMY in transgenic mice, and to explore its possible underlying molecular mechanisms. Our results *in-vitro*, *in-vivo*, and in clinical human male HCC tissues suggest that RBMY may enhance AR activity and hepatocarcinogenesis by reducing the expression of AR inhibitory variant AR45.

## Materials and Methods

### Human tissue samples

Paired tumor and non-tumor liver tissues were collected from 66 surgical HCC male patients (44 HBV-related, 10 HCV-related, 5 HBV/HCV-related, and 7 non-HBV/HCV-related) at the National Taiwan University Hospital (NTUH) in 2007. All clinical samples were obtained following approval of the Research Ethics Committee of the NTUH (NTUHREC approval No. 9361701139).

### Cell culture and transfection

The human hepatocellular carcinoma cell lines HepG2 and Hep3B were originally obtained from the Bioresource Collection and Research Center (BCRC, Taiwan). HepG2, Hep3B and Huh7 (from Dr. Hui-Lin Wu of NTUH Hepatitis Research Center) cells were grown in Dulbecco's modified Eagle's medium (DMEM) supplemented with 10% fetal calf serum (HyClone, Logan, UT). Transfections were performed using Lipofectamine (Invitrogen/Life Technologies, Carlsbad, CA) according to the manufacturer's protocol.

### RNA interference

The pSUPER-based strategy was used to knockdown RBMY expression. RBMY small hairpin RNA (shRNA) was generated by ligating three 19-nucleotide sequences specific for RBMY SRGY region (exons 7–10) into the vector (OligoEngine, Inc., Seattle, WA). The sequences were shown in Table S1. Cells were grown to 80% confluence for transfection and transfected cells were selected with 300 µg/ml geneticin.

### Semi-quantitative RT-PCR and quantitative PCR

Total RNA was extracted using RNeasy Mini kit (QIAGEN, GmbH, Hilden, Germany) and subjected to reverse transcription using SuperScript III Reverse Transcriptase (Invitrogen). The primer sequences and reaction conditions were shown in Table S1. Quantitative PCR was performed using the TaqMan Gene Expression Assay mix for target RBMY (Hs00359074_m1) or endogenous control hypoxanthine phosphoribosyl transferase 1 (Hs99999909_m1) (Applied Biosystems, Foster City, CA). Quantitative PCR for AR, AR45, IGF-1, IGFBP-3, and endogenous control S26 was performed using the MasterMix Plus for SYBR Green (Applied Biosystems). Amplification signals were detected by an ABI prism7700 or 7500 Fast Real-Time PCR System (Applied Biosystems).

### Soft agar and survival assays

For soft agar assay, 2500 cells per well in growth medium containing 0.35% agarose were placed on 6-well plates with 0.5% agarose base. After 14 days of incubation, the colonies were stained with 0.005% crystal violet at room temperature for 1 hr and counted for each plate.

For survival assay, the HepG2 cells transfected with shRNA plasmids were treated with 0.5 or 0.75 mM hydrogen peroxide for 24 hr to induce apoptosis. Cell viability was measured using the Cell Proliferation Kit I (MTT) (Roche Diagnostics, Germany). Absorbance was measured at 590/690 nm using a MRX microplate reader (Dynex Technologies, Inc., VA) and cell survival was expressed as absorbance relative to that of untreated control.

### Determination of androgen receptor CAG-repeat length in human HCC tissues

The genomic region containing the CAG trinucleotide repeat was PCR-amplified and labelled with FAM, subjected to the ABI 3700 Genetic Analyzer, and scored using the GeneMapper software (Applied Biosystems). The standard curve to calculate the CAG repeat number was based on four control samples with CAG repeat numbers ranging from 17 to 35. Sequencing analysis was performed on HCC tissues from 66 study subjects with CAG repeat numbers out of the standard curve.

### Establishment of RBMY transgenic mice and follow-up histopathology

The RBMY coding sequence was amplified and sub-cloned into the pBS-HCRHPI-A vector with a liver-specific α1-antitrypsin promoter. The construct was digested with *SpeI* to generate a transgene fragment for injection into the pro-nuclei of fertilized eggs of FVB/N mice. The Institutional Animal Care and Use Committee of the College of Medicine and College of Public Health, National Taiwan University approved the animal care and experimental procedures (IACUC approval No. 20060217).

The mice were sacrificed at 4–24 months old. Their livers were fixed in 10% neutral-buffered formalin or embedded in OCT compound (Sakura Finetek, Torrance, CA). Paraffin sections were stained with hematoxylin and eosin for histopathology examination, while frozen sections were stained with oil red O to detect fatty droplet accumulation. The severity of steatosis was estimated by the percentage of positive staining using a morphologic semi-quantitative method and classified into grade 1 (<33%), grade 2 (33–66%), or grade 3 (>66%) as described previously [27].

### Diethylnitrosamine treatment and histopathology in mice

In the chemical carcinogen model, 14-day old RBMY transgenic or control mice randomly received a single intra-peritoneal injection of diethylnitrosamine (DEN) (Sigma-Aldrich, Inc., St Louis, MO) at 10 mg/kg of body weight or saline solution of equal volume, respectively. The mice were sacrificed at 26 and 34 weeks post-treatment. In addition, earlier sacrifice at 14 weeks was specifically adopted for male mice that were reportedly highly susceptible to DEN-induced hepatocarcinogenesis. Measurements of tumor masses visible on the liver surface were recorded. Microscopic lesions were counted from two representative sections (50-µm distance) of each mouse liver.

### Immuno-histochemistry

Immuno-histochemical staining for the RBMY antigen was performed on frozen liver sections. After antigen retrieval and quenching of endogenous peroxidation, the rabbit anti-SRGY antibody [24] was incubated with sections at 1∶400 dilution overnight at 4°C. HRP-DAB was used as a detection system (R&D Systems, Inc., Minneapolis, MN) and counter-stained by hematoxylin.

### Western blotting

The extraction of total protein, Western blot analysis, and anti-SRGY antibody preparation were performed as described previously [24]. Briefly, 50 µg of total protein extracted from individual mouse liver tissue were separated by electrophoresis using standard SDS-PAGE. The blots were incubated with rabbit polyclonal anti-SRGY antibodies at 1∶5000 dilution and mouse monoclonal anti-glyceraldehyde-3-phosphate dehydrogenase (GAPDH) antibodies (Abcam, Cambridge, UK) at 1∶1000 dilution.

### Statistical analysis

Statistical differences in the association between AR-CAG repeats and RBMY, viability assay, fold change in RNA expression, and DEN treatment analysis were analyzed by two-tailed Student's *t*-test. Difference of steatosis incidences between the transgenic and control mice was analyzed by Chi-square test. Soft-agar colony assay was evaluated by one-way ANOVA followed by *t*-test. After normalization against S26, relative values of AR and AR45 expression were analyzed by Student's *t*-test with unequal variance. A *p*<0.05 was considered statistically significant, while a *p* value between 0.05 and 0.1 was considered as having a trend of difference.

## Results

### RBMY knockdown reduced the transformation and anti-apoptotic efficiencies of HepG2 cells

**Figure 1:**
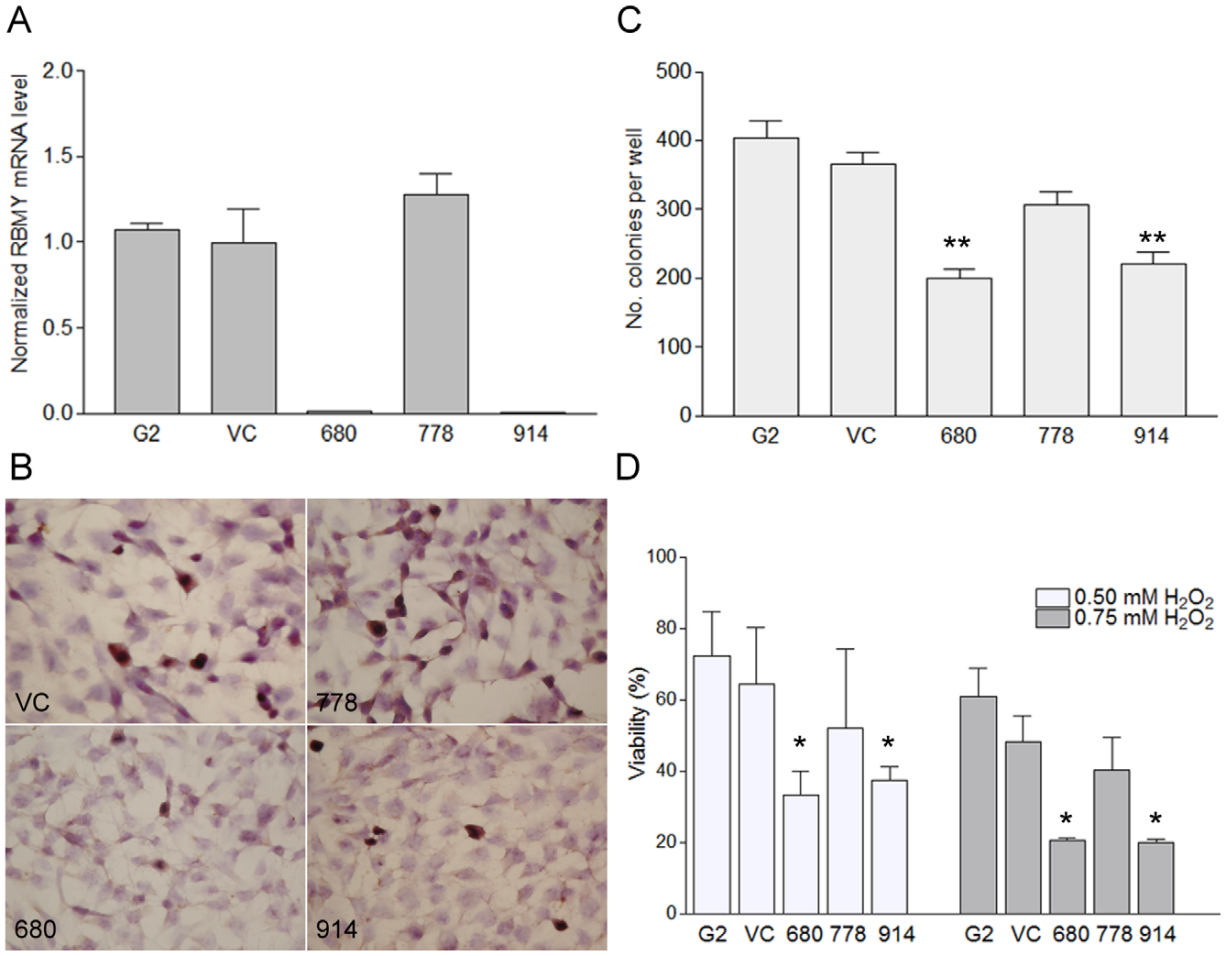
RBMY knockdown reduced the transformation and anti-apoptotic efficiencies of HepG2 cells. (**A**) Quantitative RT-PCR analysis of RBMY in parental (G2), vector plasmid (VC), pSUPER-680, pSUPER-778, and pSUPER-914 plasmids transfected HepG2 cells. (**B**) Immuno-histochemical staining of HepG2 and the corresponding transfectants for RBMY protein. (**C**) Colony formation of parental or transfected HepG2 cells on soft agar. (**D**) Percentage of cell viability by MTT assay post 0.5 or 0.75 mM hydrogen peroxide (H_2_O_2_) treatment. Data were presented as mean±SD in four independent experiments. *p<0.05; **p<0.01.

Natively expressed RBMY in human hepatoma cell line HepG2 was interfered by shRNA specifically targeting to the SRGY domain of RBMY. Quantitative RT-PCR showed that >95% of RBMY transcripts were inhibited in pSUPER-680 and pSUPER-914 transfected HepG2 cells, whereas pSUPER-778 had no knockdown effect compared to vector transfected cells (Fig. 1A). Immuno-histochemistry analysis further confirmed efficient knockdown of RBMY in pSUPER-680 and pSUPER-914 transfected cells (Fig. 1B). The vector-only transfectants expressed similar levels of RBMY compared to parental HepG2 cells and were therefore used as controls for *in vitro* cell experiments.

The anchorage independence assay showed that RBMY-expressing HepG2 cells had stronger clonogenic ability compared to RBMY knockdown cells (*p*<0.0001, one-way ANOVA). The colony numbers of pSUPER-680 and pSUPER-914 transfected cells on soft agar were reduced by 45% and 40%, respectively, compared to empty vector transfectants (Fig. 1C). Colony formation of RBMY-expressing pSUPER-778 transfectants was also significantly higher than knockdown transfectants (680 vs. 778, *p*<0.0001; 914 vs. 778, *p* = 0.001).

The anti-apoptotic abilities of RBMY-expressing and knockdown HepG2 transfectants were analyzed by survival assay after hydrogen peroxide treatment. Compared to empty vector transfectants, the viability of pSUPER-680 shRNA transfectants with 0.5 or 0.75 mM hydrogen peroxide treatment was reduced by 46% and 53% (*p*<0.05), respectively, and those of pSUPER-914 transfectants were reduced by 34% and 54% (*p*<0.05), respectively (Fig. 1D). The viability of RBMY-expressing transfectants pSUPER-778 was 16–19% lower than that of vector transfectants (*p* = 0.5).

### RBMY knockdown increased the expression of inhibitory androgen receptor variant AR45 and decreased AR trans-activation activity, while RBMY overexpression suppressed AR45 levels

**Figure 2:**
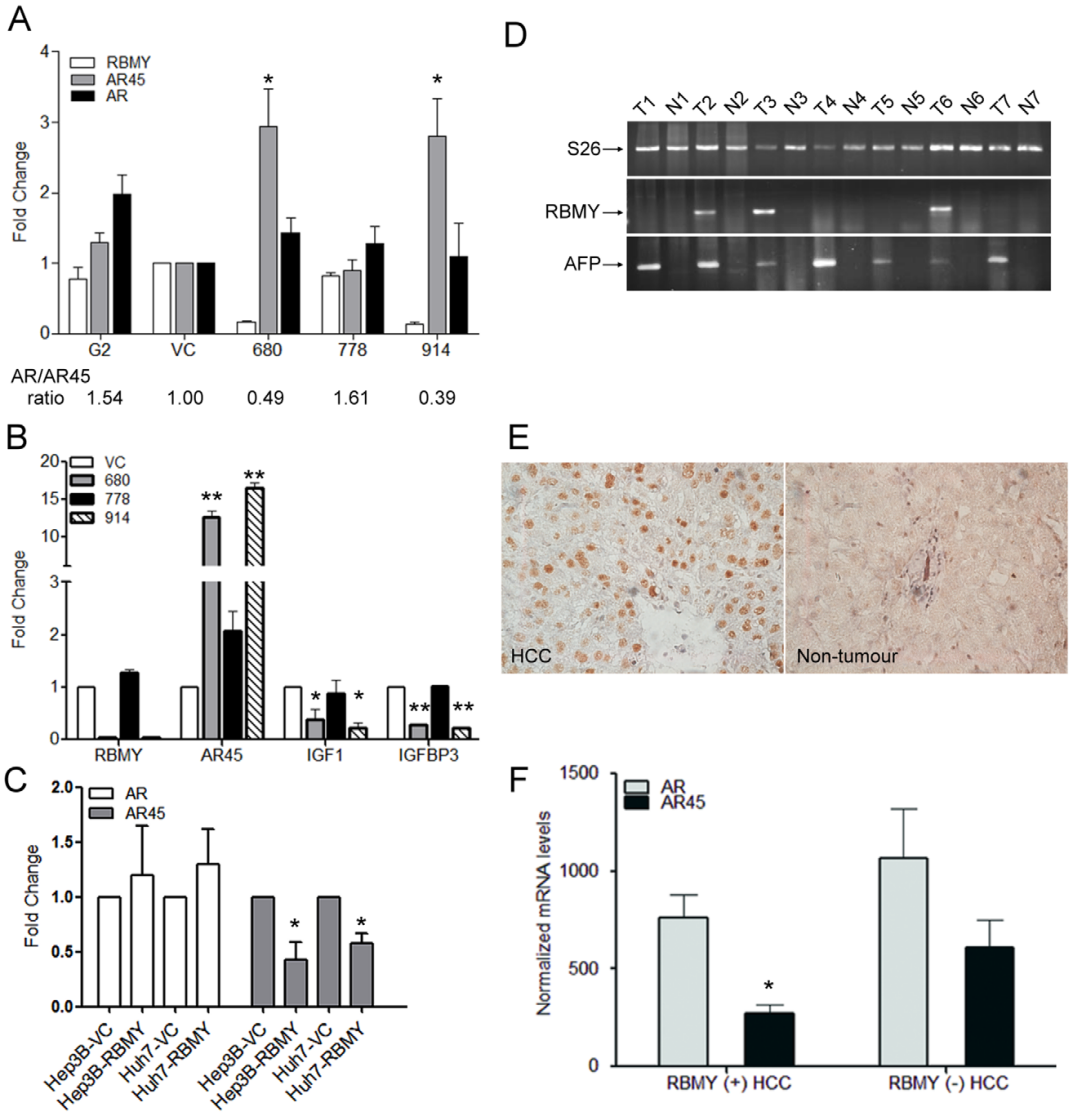
RBMY expression correlated with reduced androgen receptor AR45 variant in human hepatoma cell lines and male HCC tissues. (**A**) Semi-quantitative RT-PCR and densitometric analysis of RBMY, AR and AR45 in parental (G2), vector plasmid (VC), pSUPER-680, pSUPER-778, and pSUPER-914 plasmids transfected HepG2 cells. (**B**) Quantitative RT-PCR analysis of RBMY, AR45, IGF-1 and IGFBP-3 in RBMY knockdown transfectants (680 and 914) and non-knockdown transfectants (VC and 778). Values were normalized against the internal control S26. Data are mean±SD folds over vector control (VC). (**C**) Quantitative RT-PCR analysis of AR and AR45 in vector control or RBMY transfected Hep3B and Huh7 cells. Data are mean±SD folds over vector control (VC). (**D**) Semi-quantitative RT-PCR analysis of RBMY in the tumor (T) and non-tumor (N) parts of human male HCCs. S26 is an internal control and alpha fetoprotein (AFP) as a tumor marker. (**E**) Immuno-histochemical staining of nuclear RBMY protein in HCC tissues only (×400). (**F**) AR and AR45 mRNA expression in 7 RBMY-expressing and 5 non-expressing human male HCC tissues. The results were the mean of three different experiments. *p<0.05; **p<0.01.

It has been reported that Sam68, as an alternative splicing regulator and interacting protein of RBMY, might modulate AR-regulated transcriptional activity in prostate cancer cells [25]. To examine whether the expression ratio of AR and its inhibitory isoform AR45 were affected by RBMY, semi-quantitative RT-PCR was performed for RBMY-expressing or knockdown HepG2 cells. Densitometry analysis showed a three-fold increase of AR45 levels in RBMY knockdown transfectants (pSUPER-680 and pSUPER-914) compared to vector-only (VC) transfectants (*p*<0.05) (Fig. 2A). AR45 levels in parental HepG2 cells and pSUPER-778 transfectants were similar to those in vector transfectants. There was no significant change in AR levels between RBMY-expressing and knockdown cells. The AR/AR45 ratios in RBMY knockdown transfectants (pSUPER-680 and pSUPER-914) were reduced by 2- to 2.5-fold compared to vector-only (VC) transfectants (Fig. 2A).

As AR45 was reported to suppress AR trans-activation activity [13], the expression of AR target genes IGF-1 and IGFBP-3 was assessed and compared between RBMY-expressing and knockdown HepG2 cells. Quantitative RT-PCR analysis revealed that RBMY knockdown cells expressed high levels of AR45 but significantly reduced IGF-1 (*p*<0.05) and IGFBP-3 (*p*<0.01) levels (Fig. 2B), showing that RBMY knockdown correlated with enhanced AR45 expression and repressed AR activity.

Furthermore, the suppression of AR45 expression by over-expressing RBMY was also demonstrated in Hep3B and Huh7 cells. AR45 levels were reduced to 42% in RBMY transfected Hep3B and 60% in Huh7 cells (*p*<0.05) (Fig. 2C). There was no significant change in AR levels between RBMY-transfected and vector-transfected cells.

### RBMY expression correlated with reduced AR45 levels and shorter AR-CAG repeats in human male HCC tissues

Based on the above mentioned results showing that RBMY expression was associated with lower AR45 levels in HepG2 cells, AR and AR45 expressions were further compared in RBMY-expressing and non-expressing human male HCC tissues. Semi-quantitative RT-PCR showed that RBMY was detected in 35% (23/66) male HCC tumor tissues, which were confirmed by the expression of tumor marker alpha fetoprotein (Fig. 2D). Immuno-histochemical staining confirmed the nuclear expression of RBMY only in HCC tumor tissues (Fig. 2E).The positive rate of RBMY transcripts was 36% (16/44) in HBV-related HCC, 40% (4/10) in HCV-related HCC, 20% (1/5) in HBV/HCV-related HCC, and 29% (2/7) non-viral related HCC. There were no detectable RBMY transcripts or proteins in the non-tumor portions of the 66 HCC liver tissues.

The expression of AR and AR45 variant in seven RBMY-expressing and five non-expressing male human HCC tissues was determined by 2exp^(−ΔΔCt)^ method for relative quantification. The mRNA levels in HCC tissues were normalized against the internal control S26 and compared to the levels in vector-only HepG2 transfectants. Significantly lower levels of AR45 were detected in RBMY-expressing male HCC compared to non-RBMY expressing HCC tissues (*p*<0.05) (Fig. 2F), reflecting RBMY suppression on AR45 transcription. There was no significant difference of AR levels between RBMY-expressing and non-expressing HCC tissues.

To further assess the association between RBMY and AR activity, the CAG-repeat length of the AR gene, an AR activity-associated factor, was determined in RBMY-expressing or non-expressing male human HCC tissues. The mean CAG-repeat length in 49 HBV-related male HCC tissues (RBMY positive/negative = 17/32) was significantly shorter in those with than in those without RBMY expression (21.0±2.8 vs. 22.9±2.8, *p*<0.05). The association of CAG-repeat length and RBMY expression revealed a trend of difference (21.4±2.54 vs. 22.7±2.75, *p* = 0.064) when non-HBV HCC tissues were included (RBMY positive/negative = 23/43).

### RBMY transgenic mice developed hepatic fatty change and neoplastic lesions

**Figure 3:**
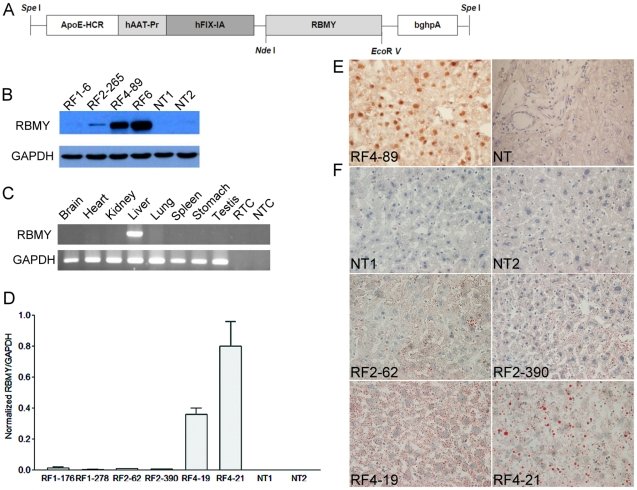
Characterization of RBMY transgenic mice. (**A**) Genetic map of the 4.2 kb RBMY transgene, including a hepatic locus control region from the apolipoprotein E gene (ApoE-HCR), liver-specific α1-antitrypsin promoter (hAAt-Pr), truncated factor IX intron (hFIX-IA), and bovine growth hormone polyadenylation signal (bghpA). (**B**) Western blot analysis of RBMY prepared from the livers of individual transgenic (RF1-6, RF2-265, RF4-89, RF6) and control (NT1, NT2) mice. (**C**) Liver-specific expression of RBMY in transgenic mice by RT-PCR using GAPDH as an internal control. Reactions without template RNA (RTC) or cDNA (NTC) were negative controls. (**D**) Expression of RBMY in transgenic (RF1-176, RF1-278, RF2-62, RF2-390, RF4-19, RF4-21) and control (NT1, NT2) mice by quantitative RT-PCR. (**E**) Immuno-histochemical staining of transgenic (RF4-89) and control (NT) mice liver tissues for RBMY. Transgenic mouse showed nuclear staining for RBMY (×400). (**F**) Oil red staining showed hepatic fatty changes in control mice (NT1 and NT2), low RBMY (RF2-62 and RF2-390) and high RBMY (RF4-19 and RF4-21) transgenic mice (×400).

Transgenic mice with liver-specific expression of RBMY were established (Fig. 3A). Western blot showed low RBMY expression in transgenic founders RF1 and RF2, but high RBMY level in RF4 and RF6 (Fig. 3B). There was no transgenic RBMY detected in the brain, heart, kidney, lung, spleen, stomach, or testis by RT-PCR (Fig. 3C), indicating a liver-specific expression of RBMY in transgenic mice. Quantitative RT-PCR results further supported RF4 as a high expression founder (Fig. 3D). IHC staining showed that RBMY was mainly located in the nucleus of transgenic mice liver tissues (Fig. 3E).

Hepatocellular changes were analyzed from liver sections of 79 transgenic mice and 30 control FVB/N mice. High RBMY-expressing RF4 founders (RF4-19 and RF4-21) displayed more severe fatty deposit than low RBMY-expressing RF2 founders (RF2-62 and RF2-390) (Fig. 3F). In RBMY transgenic mice, the incidence of developing moderate-to-severe hepatic steatosis was 60–89% (average 73%), which was ∼2.5-fold higher than that of the control group (average 30%, *p*<0.001) (Table 1). RBMY expression did not increase fibrosis or cirrhosis in the transgenic mice model.

**Table 1:** Summary of histological findings of RBMY transgenic and control mice.

Mice	Gender	Age	Liver/body	Incidence of	Incidence of
	(Mice No.)	(days)	weight	grade 2/3	pre-neoplastic lesions
			(×100)	steatosis	/adenoma/HCC
TgRBMY	F (37)	496.4±158.3	4.49±0.97	89%**	1/37
	M (42)	474.2±154.0	4.69±1.02	60%*	2/42
NT	F (15)	439.9±191.8	5.09±1.05	40%	0/15
	M (15)	433.5±185.1	5.21±1.14	20%	0/15
TgRBMY	F+M (79)	484.6±155.4	4.60±1.00	73%**	3/79
NT	F+M (30)	436.7±185.3	5.15±1.08	30%	0/30

The liver frozen sections from control (NT) and RBMY transgenic (TgRBMY) mice were stained with oil red O and classified to grade 2 and 3 with 33∼66% and >66% positive stain, respectively.

The paraffin sections were stained with hematoxylin and eosin for histopathology examination.

Values are shown as mean ± SD.

*p<0.05;

**p<0.001; transgenic vs. control mice; by Chi-square test.

**Figure 4:**
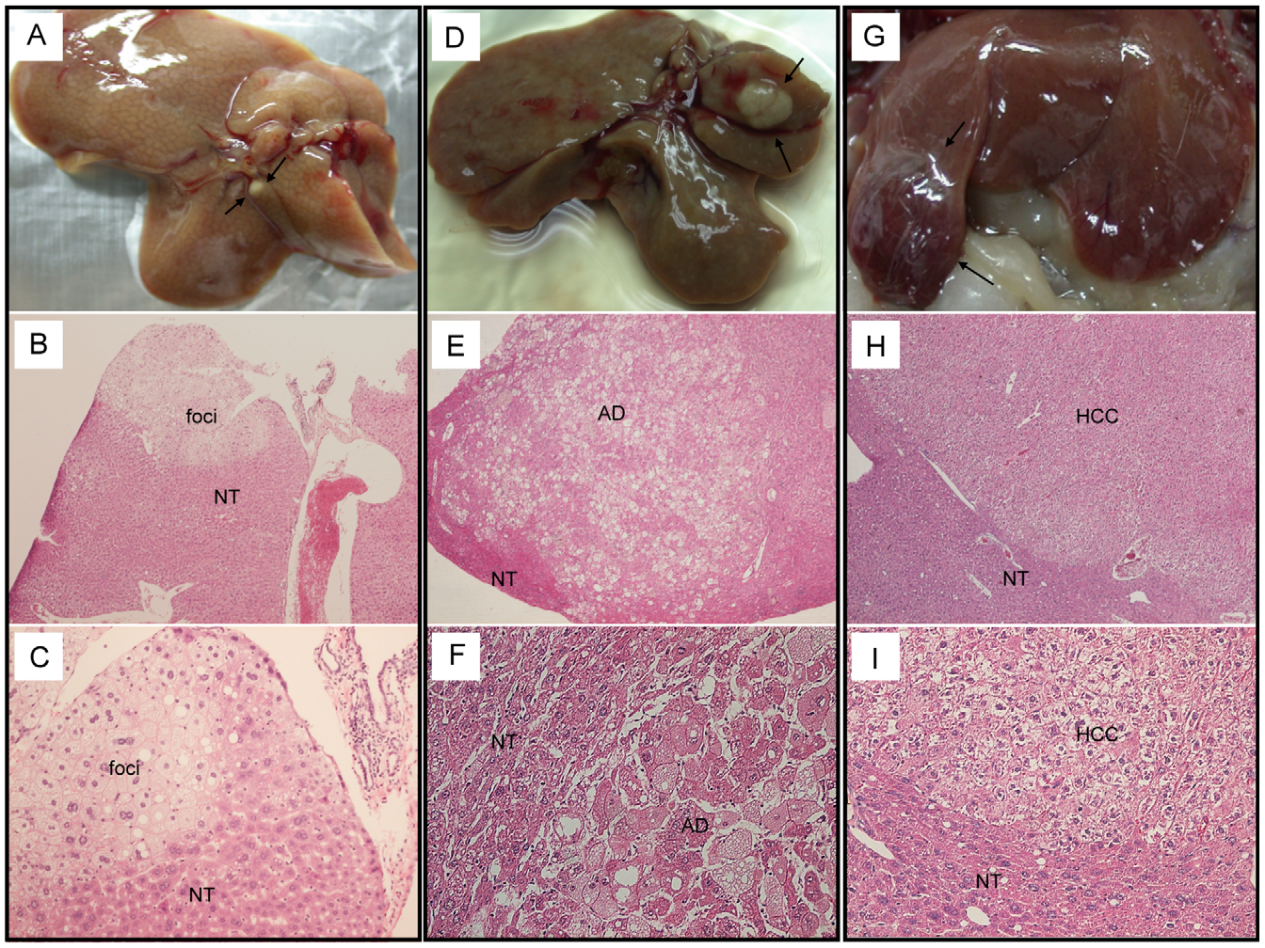
RBMY induced hepatic neoplastic changes in transgenic mice. (**A–C**) Pre-neoplastic lesion in a 15-month-old male mouse RF1-278. (**D–F**) Adenoma (AD) in a 24-month-old female mouse RF4-21. (**G–I**) HCC in a 21-month-old male mouse RF2-390. Magnification: B, E, H, ×50; C, F, I, ×200.

Among the 45 transgenic mice which were older than 15 months, three of them developed hepatic cancerous lesions (Table 1). A nodule (1 mm in diameter) was found in the right lobe of RF1-278 mouse (15 months) and histological examination revealed a pre-neoplastic lesion (Fig. 4A–C). The RF4-21 mouse (24 months) had an adenoma nodule (4 mm in diameter) in the right lobe (Fig. 4D–F) while RF2-390 (21 months) had moderately differentiated HCC (6 mm in diameter) in the median lobe (Fig. 4G–I). Medium-to-severe fatty changes were observed in the tumor parts of the three mice. None of 17 control mice older than 15 months developed hepatic cancerous lesions.

### RBMY accelerated diethyl nitrosamine-induced hepatocarcinogenesis

The tumor-promoting effects of RBMY were further assessed in the chemical carcinogenesis model. Because of the high susceptibility of male mice to DEN, male groups were sacrificed at 14 weeks post-treatment. A higher incidence of cancerous lesions was observed in transgenic male mice as compared to that in the control group (12/14 vs. 5/12, *p*<0.05). At 34 weeks post-treatment, male RBMY transgenic mice developed more tumors with diameter >3 mm compared to the control group (52 vs. 19, *p*<0.05) (Table 2). There was also a higher incidence of trabecular cancerous lesions in female transgenic mice compared to the control group at 26 weeks (7/9 vs. 1/10, *p*<0.01) and 34 weeks (9/10 vs. 3/13, *p*<0.01) post DEN treatment (Table 2).

**Table 2:** Incidence of hepatocarcinogenesis induced by DEN in RBMY transgenic and control mice.

Gender	RBMY	14 wks	26 wks	34 wks	
		Incidence of	Incidence of	Incidence of	No. tumor
		cancerous	cancerous lesions	cancerous lesions	≥3 mm
		lesions (No. mice)	(No. mice)	(No. Mice)	(No. mice)
F	+	ND	78% (7/9)**	90% (9/10)**	0 (10)
F	−	ND	10% (1/10)	23% (3/13)	0 (13)
M	+	86% (12/14)*	100% (5/5)	100% (6/6)	^52 (6)^ *
M	−	42% (5/12)	90% (9/10)	100% (7/7)	19 (7)

RBMY transgenic or control mice (14-day old) received a single intra-peritoneal injection of diethyl nitrosamine (DEN) 10 mg/kg of body weight. They were euthanized by cervical dislocation at 26 and 34 weeks for females, and at 14, 26, and 34 weeks for males after injection.

ND, not determined.

*p<0.05;

**p<0.01, RBMY-positive vs. RBMY-negative mice; by Student's *t* test.

## Discussion

RBMY is regarded as a testis-specific splicing factor in spermatogenesis [22], [23]. It has been reported to be expressed in more than one-third of human male HCC tissues [24]. This study further demonstrates that RBMY knockdown correlates with increased AR45 variant expression and reduced anchorage independent growth and anti-apoptotic abilities of human hepatoma cell line HepG2. AR45 isoform is reported to act as a dominant-negative inhibitor of AR function through the formation of AR-AR45 heterodimers [13]. We also demonstrate that RBMY knockdown reduces AR trans-activation activity in HepG2 cells. Therefore, RBMY may function as a male-specific oncogene and increase the risk of human male hepatocarcinogenesis through regulation of AR gene expression and activity.

The AR gene, rather than androgen, has been shown to play a key role in the male predominance of HCC in a transgenic HBV mouse model [17]. Human AR is composed of N terminal transactivation domain, a central DNA-binding domain, and a C terminal ligand-binding domain. AR45, a naturally occurring variant form of human androgen receptor, lacks the N-terminal domain required for full ligand activated transcriptional activity [13]. The inverse association of AR and AR45 expression has been observed in human heart and muscle tissues with the highest levels of AR45 and the lowest levels of AR [13]. In this study, RBMY knockdown increases expression of AR45 in human hepatoma HepG2 cell line, while RBMY overexpression reduces AR45 levels in Hep3B and Huh7 cell lines. Moreover, RBMY-positive human male HCC tissues express lower AR45 levels compared to RBMY-negative HCC. The down-regulation of AR target genes IGF-1 and IGFBP-3 in RBMY knockdown HepG2 cells further illustrates the enhancing effect of RBMY on AR trans-activation activity. HBV X protein has been reported to function as a virus-encoding AR co-activator that significantly contributes to the male predominance of HBV-related human HCC [16]. However, it cannot explain the gender disparity of non-HBV-related HCC. Similar ratio of RBMY expression in HBV-related, HCV-related, and non-viral-related male HCCs suggests that RBMY may be a common risk factor increasing male susceptibility for liver cancer. Our findings provide a novel mechanism interpreting the male predominance in all types of liver cancers through the Y chromosome-specific RBMY gene.

The AR45-specific exon 1B lies between the first and the second exons of the AR gene. Two hypothetical regulatory mechanisms for AR45 synthesis have been proposed, including a transcriptional control by a novel promoter upstream of exon 1B or an alternative splicing event [13]. RBMY acts as a testis-specific splicing regulator and reportedly interacts with RNA-binding protein Sam68 in the testis [26]. Sam68 is a ubiquitous splicing regulator and also a downstream target of the Src signaling pathway [28], [29]. It is thus far unclear whether or not RBMY either directly regulates AR45 transcription/splicing or via interacting with Sam68. In addition, Sam68 is considered an AR co-activator as it can modulate AR transcriptional activity in prostate cancers [25]. The Src signaling pathway also has been shown to be critical for the HBV X protein-mediated enhancement of AR function [30]. Whether RBMY is involved in the Sam68-mediated Src signaling pathway is an interesting issue to be studied in the future.

The oncogenicity of RBMY has been shown by the transformation of mouse fibroblast cell line NIH3T3 [24]. This study further demonstrates that RBMY enhances liver carcinogenesis *in vivo* in a transgenic mouse model. The reported spontaneous liver tumor growth rates are 3% and 0% in 24 month-old male and female wild type FVB/N mice, respectively [31]. RBMY transgenic mice developed liver tumors in 8.7% (2/23) of male and 4.5% (1/22) of female mice older than 15 months, which are more than 2-fold higher than the spontaneous liver tumor incidences.

In the model of DEN-induced hepatocarcinogenesis, the significance of RBMY enhancing effect on cancerous lesion formation is observed as early as 14 weeks post-treatment in male transgenic mice. In addition, larger tumors (diameter ≥3 mm) developed in transgenic male mice at 34 weeks, indicating RBMY enhancement on DEN-induced hepatocarcinogenesis. Even female RBMY transgenic mice also have significantly increased incidence of cancerous lesions at both 26 and 34 weeks post-treatment. Although the wild type female mice are resistant to DEN carcinogenesis due to the protective effect of estrogen-mediated inhibition of IL-6 production by Kupffer cells [32], [33], the results here demonstrate the delivery and activation of a male-specific RBMY gene accelerated liver cancer development even in female transgenic mice.

In conclusion, RBMY knockdown elicits inhibitory effects on the transformation and anti-apoptotic abilities of the human hepatoma cell line HepG2. The inhibitory effect of RBMY on AR45 levels is demonstrated in RBMY knockdown HepG2 cells and RBMY over-expressing Hep3B and Huh7 cells. Therefore, the oncogenic mechanism of RBMY may be linked to its regulation of AR trans-activation activity by the increase of AR45 variant, the inhibitor of AR. AR45 expression detected in RBMY-expressing human male HCC is also significantly lower compared to non-expressing HCC tissues. Furthermore, RBMY exhibits tumor-promoting activity *in vivo* in a transgenic mice model and accelerates the development of neoplastic lesions in a diethylnitrosamine-induced hepatocarcinogenesis animal model. The carcinogenic effect in hepatoma cell line and human HCC tissues, along with *in vivo* tumor promotion in the transgenic mice, provides a novel role of RBMY as a male-specific oncogene to explain the male predominance of liver cancer.

## Supporting Information

Table S1 **Primer sequences in shRNA plasmid construction, semi-quantitative and real-time RT-PCR.**
(DOC)
