# Correction: Male Germ Cell-Specific RNA Binding Protein RBMY: A New Oncogene Explaining Male Predominance in Liver Cancer

**DOI:** 10.1371/annotation/3a796c69-0e20-472e-b1ae-70d473e1e072

**Published:** 2012-01-27

**Authors:** Daw-Jen Tsuei, Po-Huang Lee, Hsiao-Yu Peng, Hsiao-Ling Lu, De-Shiuan Su, Yung-Ming Jeng, Hey-Chi Hsu, Shu-Hao Hsu, Jia-Feng Wu, Yen-Hsuan Ni, Mei-Hwei Chang

There was an error in the fourth author's name. The correct spelling is Hsiao-Ling Lu. The correct citation is: Tsuei D-J, Lee P-H, Peng H-Y, Lu H-L, Su D-S, et al. (2011) Male Germ Cell-Specific RNA Binding Protein RBMY: A New Oncogene Explaining Male Predominance in Liver Cancer. PLoS ONE 6(11): e26948. doi:10.1371/journal.pone.0026948 

