# Supplementary material for: Male Germ Cell-Specific RNA Binding Protein RBMY: A New Oncogene Explaining Male Predominance in Liver Cancer
Source: PLoS One. 2011 Nov 4;6(11):e26948. doi: 10.1371/journal.pone.0026948 (PMC3208560; doi:10.1371/journal.pone.0026948)
Supplement: Table S1 — Primer sequences in shRNA plasmid construction, semi-quantitative and real-time RT-PCR. (DOC) [file pone.0026948.s001.doc]

| **Table S1.** Primer sequences in shRNA plasmid construction, semi-quantitative | | |  |
| --- | --- | --- | --- |
| and real-time RT-PCR | | |  |
| Primer |  | Sequence | |
| pSUPER-680^a^ | sense: | GATCCCCGAAACGAGGGATTATGCTC | |
| (nt 796-814) |  | ttcaagagaGAGCATAATCCCTCGTTTCTTTTTA | |
|  | antisense: | AGCTTAAAAAGAAACGAGGGATTATGCTC | |
|  |  | tctcttgaaGAGCATAATCCCTCGTTTCGGG | |
| pSUPER-778^a^ | sense: | GATCCCCTCGAAGTTCCCGAGAAACT | |
| (nt 894-912) |  | ttcaagagaAGTTTCTCGGGAACTTCGATTTTTA | |
|  | antisense: | AGCTTAAAATCGAAGTTCCCGAGAAACT | |
|  |  | tctcttgaaAGTTTCTCGGGAACTTCGAGGG | |
| pSUPER-914^a^ | sense: | GATCCCCTGCTCCACCATCTAGAGGC | |
| (nt 921-939) |  | ttcaagagaGCCTCTAGATGGTGGAGCATTTTTA | |
|  | antisense: | AGCTTAAAATGCTCCACCATCTAGAGGC | |
|  |  | tctcttgaaGCCTCTAGATGGTGGAGCAGGG | |
| RBMY^b^ | sense: | TGGCTTCCCTCACATGAAG | |
| (164-bp) | antisense: | TTGCTTCTTGCCACAGCAGAAG | |
| AFP^b^ | sense: | GTTGCCAACTCAGTGAGGAC | |
| (240-bp) | antisense: | GAGCTTGGCACAGATCCTA | |
| S26^b^ | sense: | CCGTGCCTCCAAGATGACAAAG | |
| (300-bp) | antisense: | GTTCGGTCCTTGCGGGCTTCAC | |
| AR45^c^ | sense: | TACAGGGAACCAGGGAAACGAAT | |
| (466-bp) | antisense: | CATAGCCTTCAATGTGTGACACT | |
| AR^c^ | sense: | GGGTGAGGATGGTCTCCCC | |
| (506-bp) | antisense: | CTGGACTCAGATGCTCCAAC | |
| AR45^d^ | sense: | TCCTCGGAGGTCATCTGTTC | |
| (120-bp) | antisense: | AGGAGCACTCTGCATTCGTT | |
| IGF-1^d^ | sense: | TTGAATTGAGCACCTCAAGCAT | |
| (155-BP) | antisense: | AGAAGTGCCATCTTGGGAAGAG | |
| IGFBP-3^d^ | sense: | GCCGCGGGCTCTGCGTCAACGC | |
| (415-bp) | antisense: | CTGGGACTCAGCACATTGAGGAAC | |
| ^a^Oligomers (each 3.3 ug) added into anneal buffer (100 mM NaCl, 50 mM HEPES) | | | |
| , boiled, and naturally cooled, then digested with HindIII/BglII and cloned into | | | |
| pSUPER vector. The siRNA sequence against RBMY was underlined. | | | |
| ^b^PCR for 34 cycles of 30 s at 94°C, 30 s at 50°C, 1 m at 72°C; 21 cycles for S26. | | | |
| ^c^Semi-quantitative PCR for 34 cycles of 1 m at 94°C, 1 m at 55°C, 1 m at 72°C. | | | |
| ^d^Quantitative PCR for 10 m at 95°C and 40 cycles of 15 s at 95°C, 1 m at 60°C. | | | |
